# The Lactate Nexus: A Molecular Bridge Linking Physical Activity, Sleep, and Cognitive Enhancement

**DOI:** 10.3390/biomedicines14010253

**Published:** 2026-01-22

**Authors:** Alimjan Ablitip, Kefeng Zheng, Hao Ding, Yicong Cui, Xindong Ma, Yanwei You

**Affiliations:** 1 Division of Sports Science & Physical Education, Tsinghua University, Beijing 100084, China; almjablt22@mails.tsinghua.edu.cn (A.A.); zhengkf24@mails.tsinghua.edu.cn (K.Z.); dingh22@tsinghua.org.cn (H.D.); cyc23@mails.tsinghua.edu.cn (Y.C.); 2 IDG/McGovern Institute for Brain Research, Tsinghua University, Beijing 100084, China; 3 Saw Swee Hock School of Public Health, National University of Singapore, Singapore 117549, Singapore

**Keywords:** lactate, physical activity, sleep, cognitive function

## Abstract

Physical activity (PA) and quality sleep are essential for cognitive health, providing synergistic protection against age-related cognitive decline. However, the shared molecular pathways that explain their combined and interactive benefits remain poorly understood. This review suggests that lactate, long dismissed as a metabolic waste product, is a unifying mechanism. We introduce the “Lactate Nexus”, a conceptual framework that proposes lactate functions as a key signalling molecule, mechanistically linking the pro-cognitive effects of both daytime exercise and nighttime sleep. We begin by outlining lactate’s evolving role—from an energy substrate shuttled from astrocytes to neurons (the Astrocyte–Neuron Lactate Shuttle) to a pleiotropic signal. As a signal, lactate influences neuroplasticity via NMDA receptors, neuroinflammation via the HCAR1 receptor, and gene expression through the epigenetic modification of histone lactylation. We then compile evidence demonstrating how PA provides a substantial lactate signal that activates these pathways and primes the brain’s metabolic infrastructure. Crucially, we integrate this with proof that lactate levels naturally increase during slow-wave sleep to support memory consolidation and glymphatic clearance. The “Lactate Nexus” framework offers a comprehensive molecular explanation for the synergy between PA and sleep, positioning lactate as a key signalling mediator and a promising biomarker and therapeutic target for fostering lifelong cognitive resilience.

## 1. Introduction

Dementia, the decline in cognitive function, has emerged as a principal challenge for both individual well-being and global public health, as 55 million people are living with dementia worldwide, which is expected to triple by 2050 [1,2]. Cognitive function, which encompasses memory, executive function, and processing speed, is the foundation of our ability to learn, reason, and engage meaningfully with the world. However, the degenerative changes that can accompany ageing, along with the rising prevalence of neurodegenerative conditions such as Alzheimer’s disease, pose a significant threat to this foundation. The immense societal and economic burden of cognitive decline underscores the urgent need to identify practical, accessible strategies to promote cognitive vitality and brain resilience throughout the lifespan.

Lifestyle factors are crucial for maintaining brain health and healthy ageing, particularly physical activity and adequate sleep [3]. A vast body of evidence demonstrates that regular exercise enhances neurogenesis and synaptic plasticity, improves cerebral blood flow, and significantly lessens the risk of cognitive impairment and dementia [4,5,6]. Similarly, quality sleep is indispensable for cognition, playing a critical and active role in the consolidation of memories, the homeostatic regulation of synapses, and the clearance of neurotoxic waste products from the brain [7]. While these two factors independently confer powerful cognitive benefits, they also work synergistically, with hypotheses suggesting that sleep plays a mediating and moderating role in the effect of exercise on cognitive function [3,5]. Yet, despite this strong behavioural and synergistic link, the shared molecular pathways that might unify their pro-cognitive effects remain poorly understood. The search for a common mechanistic language between these two distinct physiological states is a critical frontier in neuroscience.

This review proposes lactate as a candidate for this unifying mechanism. Historically dismissed as a metabolic waste product and a primary cause of exercise-induced fatigue, lactate has undergone a profound re-evaluation [8]. Groundbreaking research has repositioned it from a simple byproduct to a vital energy substrate and, most importantly, a sophisticated signalling molecule that communicates metabolic information between cells and tissues.

Recent reviews have expertly detailed the molecular links between exercise and cognitive health, with a strong focus on lactate as a key mediator [9,10,11,12,13]. However, a significant gap remains in understanding how lactate might unify the cognitive benefits of both physical activity and sleep. This review aims to bridge that gap by presenting the following central thesis: Lactate is a primary signalling molecule that mechanistically links the benefits of exercise and sleep to cognitive enhancement. We will argue that lactate function as a pivotal molecular link that translates the metabolic events of both daytime exertion and nighttime restoration into lasting improvements in brain plasticity and resilience.

Historically dismissed as a metabolic waste product, lactate has undergone a profound re-evaluation. Recent breakthroughs have repositioned it from a simple byproduct to a vital energy substrate and a sophisticated signalling molecule that communicates metabolic information across tissues. While current literature expertly details the molecular links between exercise and cognitive health, a significant gap remains in understanding how lactate might unify the benefits of both physical activity and sleep. The “Lactate Nexus” model proposed here suggests that lactate serves as an integrative molecular integrator, translating the metabolic events of daytime exertion and nighttime restoration into lasting improvements in brain plasticity. Therefore, the purpose of this review is to examine the evidence for lactate’s multifaceted roles and establish it as the primary mechanistic bridge linking daytime activity and nocturnal sleep to cognitive enhancement.

## 2. The Evolving Role of Lactate in Brain Energetics and Signalling

For much of the 20th century, glucose was regarded as the brain’s obligatory physiological energy substrate. At the same time, lactate was dismissed as a metabolic waste product and an indicator of oxygen debt. However, a paradigm shift over the last few decades has repositioned lactate as a central and sophisticated player in brain function [14]. It is not only a preferred energy substrate for neurons but also a dynamic signalling molecule that orchestrates a wide range of cerebral processes [15].

### 2.1. The Astrocyte–Neuron Lactate Shuttle (ANLS) Hypothesis

The classical view of brain metabolism held that glucose from the bloodstream directly fuels neurons. While this is not incorrect, it is incomplete. Over the past two decades, cell-resolution research methods have enabled the identification of cell-specific glucose metabolism in the brain, revealing that different cell types exhibit distinct metabolic characteristics. The current academic consensus holds that neurons primarily employ oxidative phosphorylation. In contrast, glial cells (particularly astrocytes and oligodendrocytes) predominantly process glucose via glycolysis, generating lactate and pyruvate [16,17]. Specifically, the study found that neurons express lactate dehydrogenase 1 (LDH_1_; present in tissues that consume lactate, such as cardiac muscle), while LDH_5_ (primarily expressed in tissues that produce lactate, such as skeletal muscle) is exclusively found in astrocytes [18]. Pyruvate kinase M2 (PKM2) and lactate dehydrogenase 5 (LDH_5_) are predominantly expressed in astrocytes, while 6-phosphofructokinase 1 (PFK-1) exhibits higher activity in astrocytes, indicating enhanced glycolytic capacity. In contrast, PKM1 and LDH_1_ are primarily expressed in neurons, with higher pyruvate dehydrogenase (PDH) activity [19].

The Astrocyte–Neuron Lactate Shuttle (ANLS) hypothesis, first proposed by Pellerin and Magistretti, provided a more nuanced model that revolutionised our understanding of neuroenergetics [20]. This model suggests a metabolic partnership between astrocytes and neurons, indicating that neurons can utilise extracellular lactate as a necessary supplement to their energy metabolism substrates. In contrast, astrocytes provide small-molecule energy substances to the neurons surrounding them by releasing lactate. Specifically, during periods of high neuronal activity, the neurotransmitter glutamate is released into the synapse. Astrocytes surrounding the synapse then take up this glutamate by excitatory amino acid transporters, EAATs, and convert glutamate to glutamine [20]. This energy-consuming process would further stimulate the astrocytes to increase their own glucose uptake via GLUT1 from nearby capillaries [21]. Inside the astrocyte, glucose is rapidly processed through aerobic glycolysis—the conversion of glucose to lactate even in the presence of oxygen. This newly produced lactate is then shuttled out of the astrocyte via monocarboxylate transporters (primarily MCT_1_ and MCT_4_) and into the extracellular space. Neurons that express high levels of the lactate transporter efficiently take up lactate. Inside the neuron, lactate is converted to pyruvate and fed directly into the mitochondrial tricarboxylic acid (TCA) cycle, generating ATP far more rapidly and efficiently than if the neuron had started with glucose. This shuttle is particularly crucial during intense cognitive tasks, where neurons require a rapid supply of energy that glucose metabolism alone may be too slow to provide [22]. By positioning astrocytes as metabolic intermediaries, the ANLS ensures that energy supply is tightly coupled to neuronal demand, making lactate a cornerstone of brain energy dynamics.

### 2.2. Beyond Fuel: Lactate as a Signalling Molecule

While its role as an energy substrate is fundamental, the most exciting recent discoveries concern lactate’s function as a signalling molecule [15]. Acting well beyond simple energy provision, lactate serves as a biochemical messenger that directly influences neuronal behaviour, vascular dynamics, and the brain’s transcriptional landscape.

At the level of synaptic plasticity, lactate is recognised as a critical facilitator of neuronal communication [23,24,25,26]. Since the importance of glycogenolysis in memory consolidation was discovered [27], several studies have investigated the role of lactate in this process, showing that the transfer of lactate from astrocytes to neurons can facilitate long-term potentiation (LTP) and is necessary for memory consolidation [24,28]. Further analysis revealed that the effect of lactate on memory does not rely on energy adaptation, but rather on enhancing the function of the N-methyl-D-aspartate (NMDA) receptor as a co-agonist or modulator. By augmenting NMDA-mediated calcium influx and triggering the expression of plasticity-related immediate-early genes, such as Arc and zif268, lactate provides the necessary biochemical infrastructure for synaptic reorganisation [29,30]. Furthermore, lactate can exert a homeostatic influence; under conditions of high metabolic stress, it can help reduce neuronal excitability by acting on ATP-sensitive potassium channels, thereby conserving energy and protecting against excitotoxicity [31].

In addition to neuronal signalling, lactate is a primary orchestrator of neurovascular coupling, ensuring that local blood flow meets the brain’s metabolic demands. The brain must precisely match blood flow, and thus oxygen and substrate delivery, to active regions. This process is known as neurovascular coupling [32]. Lactate is a key signalling agent in this dialogue between neurons and blood vessels. Lactate released from astrocytes can act on smooth muscle cells in cerebral arteries, leading to vasodilation and increased local blood flow [33]. Significantly, lactate-mediated vasodilation operates through a distinct mechanism from that of vasodilation caused by metabolic acidosis, which involves the accumulation of H+ ions. While acidosis typically promotes vasodilation by influencing potassium channel conductance or calcium sensitivity in smooth muscle cells, lactate exerts active control through specific molecular pathways. One primary mechanism involves the inhibition of the prostaglandin transporter (PGT) by extracellular lactate. By slowing the clearance of prostaglandin E_2_ (PGE_2_) from the interstitial space, lactate ensures that this potent vasodilator remains active at the astrocyte end-feet, thereby increasing local cerebral blood flow in response to neural activity. Furthermore, lactate signals through its G-protein-coupled receptor, HCAR1, expressed on perivascular cells, thereby modulating vascular tone and permeability. On a longer timescale, lactate stimulates the expression of Vascular Endothelial Growth Factor (VEGF), a potent signalling protein that promotes angiogenesis, allowing the brain to structurally adapt to sustained periods of high activity [34].

**Figure 1:**
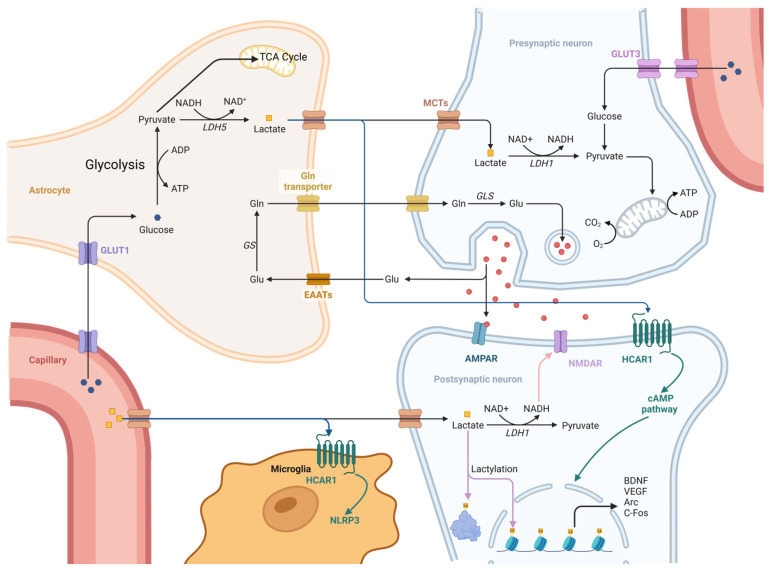
Lactate as energy substrate and signal molecule in the brain (Created in BioRender. Ablitip, A. (2026) https://BioRender.com/3v49dnz (accessed on 20 January 2026)).

A definitive piece of evidence for lactate’s signalling role was the discovery of its dedicated G-protein-coupled receptor, the hydroxycarboxylic acid receptor 1 (HCAR1), also known as GPR81, which is widely expressed on neurons and other brain cells [35,36]. When lactate binds to HCAR1, it initiates intracellular signalling cascades that are often neuroprotective, including the modulation of pathways, such as ERK1/2, NLRP3, NF-κB and Wnt [37,38,39,40,41]. Parallel to receptor-mediated signalling, lactate can also signal by being transported through neuronal MCTs and converted to pyruvate by LDH, which results in the formation of NADH and ATP [42]. The metabolic state of a cell is fundamentally reflected in its redox state, particularly the ratio of nicotinamide adenine dinucleotide in its oxidised and reduced forms. The enzymatic conversion of lactate to pyruvate (and vice versa) is directly tied to this ratio [33]. Consequently, the intracellular lactate/pyruvate ratio serves as a rapid and sensitive barometer of the cell’s metabolic and redox status [29]. This ratio can influence the activity of numerous redox-sensitive enzymes and transcription factors, thereby translating the cell’s metabolic state directly into functional and even genetic responses (Figure 1).

This diagram demonstrates lactate’s multifunctional role as both a vital energy source and a pleiotropic signalling molecule. Metabolic Pathway (The Astrocyte–Neuron Lactate Shuttle): During neuronal activity, astrocytes absorb Glucose (via GLUT1) and Glutamate (Glu) (via EAATs), stimulating glycolysis to produce and export Lactate (via LDH_5_ and MCTs). Neurons then absorb this lactate (via MCTs) and convert it to pyruvate (via LDH_1_) to support mitochondrial ATP generation. The associated glutamate-glutamine (Gln) cycle (via GS and GLS) restores the neuronal glutamate supply. Signalling Pathways: Beyond its role as a fuel, lactate functions as a key signalling molecule. Inside cells, its conversion to pyruvate (via LDH_1_) changes the NAD+/NADH redox balance, providing the substrate for Lactylation of histones, which enhances the expression of plasticity-related genes (e.g., BDNF, VEGF). Outside cells, lactate binds to the HCAR1 receptor on neurons (activating the cAMP pathway) and microglia (modulating the NLRP3 inflammasome), affecting neuroplasticity and inflammation.

Beyond its role as a metabolic intermediate, lactate is an essential regulator of mitochondrial physiology, critical for maintaining the high energy demands of cognitive processes during both physical exertion and sleep. Firstly, lactate is recognised as a preferred mitochondrial fuel for neurons; it enters the tricarboxylic acid (TCA) cycle more rapidly than glucose, thereby providing an efficient source of adenosine triphosphate (ATP) to support immediate synaptic activity [43,44,45]. Secondly, lactate acts as a potent signalling molecule that promotes mitochondrial biogenesis. It activates the Sirt1/PGC-1α signalling pathway, which regulates the expression of nuclear-encoded mitochondrial genes and enhances the oxidative capacity of hippocampal neurons [46,47]. Importantly, recent research indicates that lactate can directly stimulate the mitochondrial electron transport chain (ETC) independently of its metabolic conversion, serving as an acute signal to maintain cellular bioenergetic homeostasis under metabolic stress [48]. These diverse mitochondrial effects ensure that the brain can adapt its energetic machinery to sustain the resource-intensive processes of synaptic reorganisation and memory consolidation.

Finally, an emerging frontier in lactate research is its role in epigenetic regulation through histone lactylation. In this process, lactate modifies histone lysine residues (e.g., H3K18lac), potentially altering chromatin structure to modulate transcriptional activity [49]. While research has confirmed the presence of histone lactylation in neural cells and its responsiveness to neuronal excitation [50]. Its specific contribution to long-term memory remains an area of active investigation. This mechanism provides a radical new perspective on how transient metabolic signals might be converted into lasting changes in the brain’s transcriptional landscape. Furthermore, the post-translational modification of non-histone proteins by lactate represents a novel and promising perspective that further broadens the scope of the “Lactate Nexus” framework [51].

### 2.3. Age-Related Alterations in Brain Lactate Metabolism

The relationship between lactate and cognitive health must be considered in the context of ageing, as the production, transport, and signalling efficacy of lactate in the central nervous system (CNS) decline significantly over time. Evidence indicates that ageing is associated with a progressive depletion of astrocytic glycogen stores, the brain’s primary energy reserve, and a reduction in the activity of key glycolytic enzymes, such as phosphofructokinase and LDH. These changes collectively limit the brain’s metabolic flexibility, reducing its capacity for on-demand lactate mobilisation during periods of high neural demand [52,53,54,55]. Furthermore, the expression of monocarboxylate transporters (MCTs) exhibits a progressive, region-specific decline with advancing age. Specifically, the reduction in MCT1 in brain endothelial cells impairs the blood–brain barrier’s capacity to uptake peripherally produced lactate during physical activity. Additionally, its decrease in the oligodendrocyte lineage undermines metabolic support for ageing axons. Concurrently, the downregulation of neuronal MCT2, which is often concentrated at postsynaptic densities, hinders the efficient utilisation of astrocytic lactate for synaptic plasticity and memory consolidation [56,57,58,59,60]. These age-related metabolic shifts imply a disruption in the Lactate Nexus. In the ageing brain, daytime physical activity may not effectively stimulate the brain owing to compromised lactate transport. At the same time, nocturnal slow-wave sleep may lack the lactate flux essential for glymphatic clearance and memory replay. Such an energetic and signalling deficiency establishes a vulnerability window, which could potentially expedite the progression from healthy ageing to neurodegenerative diseases marked by chronic neuroinflammation and synaptic deterioration.

### 2.4. The Dual Nature of Lactate: From Physiological Signalling to Pathological Stress

While the “Lactate Nexus” framework primarily emphasises the pro-cognitive benefits of lactate, a comprehensive understanding requires a balanced consideration of its potential detrimental effects under extreme or pathological conditions. The biological impact of lactate is fundamentally determined by its concentration, the concomitant pH environment, and the specific physiological context. In pathological states such as ischemic stroke, traumatic brain injury (TBI), or status epilepticus, lactate can accumulate to concentrations exceeding 20 mM. Such excessive accumulation is often associated with severe intracellular acidosis and the overactivation of acid-sensing ion channels (ASICs), leading to neuronal apoptosis and blood–brain barrier disruption [61,62,63]. These findings underscore that the neuroprotective role of lactate is predicated on its homeostatic maintenance; once the concentration surpasses a critical physiological threshold, the metabolic burden and pH-driven toxicity may override its signalling benefits.

Beyond acute pathological states, the ceiling effect of lactate-mediated benefits is also evident at higher exercise intensities. The relationship between physical activity and cognitive function is widely recognised as following an inverted U-shaped curve, where moderate-to-high intensity exercise optimises cognitive gains [64,65]. However, excessive vigorous exercise may reach a tipping point where the systemic and central stress responses begin to impair rather than enhance cognition [66,67,68].

This emerging evidence suggests that the Lactate Nexus operates within a refined metabolic window. Within this window, exercise-induced lactate surges act as a precision signal for plasticity; however, when the physiological limit is exceeded, either through pathological accumulation or excessive physical exertion, competing detrimental mechanisms, such as mitochondrial vesicle-mediated neuroinflammation, may become dominant. By delineating these boundaries, it becomes clear that the cognitive enhancement facilitated by the Lactate Nexus is a product of regulated metabolic flux rather than an indiscriminate increase in lactate levels.

## 3. Physical Activity: Priming the Brain with Lactate

Physical activity is widely recognised as a key non-pharmacological intervention for promoting cognitive health and combating neurodegenerative diseases. Numerous epidemiological studies have confirmed that both leisure-time physical activity and the amount of exercise recommended by the World Health Organisation are closely associated with reduced depressive symptoms, a lower risk of metabolic syndrome, and improved cognitive performance [69,70]. High-intensity interval training (HIIT) and other high-performance exercise modalities have become a research hotspot, particularly for their health-promoting effects on inflammation [71,72]. While the benefits of physical activity for the brain are readily apparent, the underlying molecular mechanisms—particularly how mechanical muscle movement is translated into enhanced brain neuroplasticity—remain central to current neuroscience research. The link between regular physical activity and enhanced cognitive function is unequivocally established. Exercise improves memory, sharpens executive functions, and provides a powerful buffer against age-related mental decline and neurodegenerative diseases. While these benefits are multifaceted, a growing body of evidence suggests that lactate is a primary molecular mediator that translates muscle activity into enhanced brain plasticity. This chapter outlines the mechanisms by which exercise increases lactate levels in the brain and examines the key pathways through which this lactate signal promotes cognitive health.

### 3.1. Exercise-Induced Lactate Production and Brain Uptake

Physical exercise, particularly at moderate to high intensities, fundamentally alters the body’s metabolic landscape, leading to a dramatic increase in brain lactate availability from two synergistic sources: peripheral production and central production. First, during strenuous activity, skeletal muscles engage in high rates of glycolysis, producing substantial amounts of lactate that are released into the bloodstream. This can elevate arterial lactate concentrations from a resting level of ~1–2 mM to levels of up to 20 mM during exhaustive exercise [67]. This circulating, muscle-derived lactate acts as an endocrine-like signal, readily crossing the blood–brain barrier via monocarboxylate transporter 1 (MCT1), which is highly expressed on the endothelial cells of brain capillaries. The brain’s uptake of lactate is directly proportional to its arterial concentration. It does not appear to saturate even at very high levels, demonstrating the brain’s high capacity for lactate transport [44]. Second, exercise simultaneously increases central, brain-derived lactate production. The heightened neuronal activity associated with motor control and sensory processing during exercise stimulates the release of neurotransmitters, such as glutamate [73]. This triggers astrocytes to increase their metabolic activity in two ways: by breaking down their glycogen stores (glycogenolysis) and by enhancing their glucose uptake and metabolism. Both processes culminate in the production and release of lactate into the local neural environment, representing a paracrine signalling mechanism [45]. Together, these peripheral and central pathways ensure that physical activity provides a robust, multifaceted lactate signal to the brain, serving as both a supplemental energy source and a potent signalling molecule.

### 3.2. Lactate-Mediated Mechanisms of Cognitive Enhancement

While the molecular capacity of lactate is intrinsic, physical activity serves as the primary physiological engine that activates these signalling pathways in a dose-dependent manner. The massive surge of systemic lactate during exercise—often reaching concentrations of 10–20 mM—creates a substantial gradient that drives a huge influx into the central nervous system via the monocarboxylate transporter (MCT) system. This acute lactate shock is a unique feature of physical exertion, initiating a cascade of neurotrophic, anti-inflammatory, and epigenetic events that underpin cognitive enhancement.

#### 3.2.1. Neurotrophic Factors and Plasticity

Exercise-induced lactate is a powerful driver of neuroplasticity. A key mechanism is its ability to stimulate the expression of Brain-Derived Neurotrophic Factor (BDNF), a master regulator of synaptic health. While baseline lactate levels support metabolic homeostasis, the heightened levels generated during exercise are specifically required to induce the hippocampal Sirtuin1/PGC-1α/FNDC5 signalling pathway. This exercise-specific cascade promotes both the synthesis of BDNF and the expression of its receptor, TrkB, creating an environment conducive to learning and memory [74,75].

Furthermore, lactate directly facilitates cellular processes of memory formation by potentiating NMDA receptor activity [29]. This enhances calcium influx into neurons, a critical step in inducing Long-Term Potentiation (LTP) and triggering the expression of plasticity-related immediate-early genes (IEGs), such as Arc, c-Fos, and Zif268 [29,76]. Evidence suggests that this metabolic legacy is not transient; acute bouts of exercise have been shown to upregulate MCT1 and MCT2 expression, with this effect persisting for up to 24 h, effectively overlapping with subsequent sleep cycles [77]. This ensures that the brain possesses a heightened capacity for lactate uptake and utilisation during the restorative phases of sleep. By promoting both the key growth factor (BDNF) and the core synaptic machinery (NMDA receptors, IEGs), exercise-derived lactate creates an optimal environment for robust learning and memory.

#### 3.2.2. Modulation of Neuroinflammation

Chronic, low-grade neuroinflammation, often mediated by overactive microglial cells, is detrimental to cognitive function and is a hallmark of brain ageing [78]. Exercise-induced lactate has demonstrated potent anti-inflammatory properties by activating the HCAR1 receptor, which in turn suppresses the NLRP3 inflammasome pathway [79]. This activation helps shift microglia from a pro-inflammatory state to an anti-inflammatory, neuroprotective phenotype. This reduction in the brain’s inflammatory tone creates a more permissive environment for neurogenesis, synaptic plasticity, and overall neuronal health, thereby counteracting a key driver of cognitive decline.

This mechanism’s systemic impact is supported by epidemiological evidence showing that physical activity is negatively correlated with inflammatory markers, including C-reactive protein (CRP) and the systemic immune-inflammatory index (SII). These anti-inflammatory effects of exercise are particularly pronounced in individuals with insufficient sleep, where lactate may act as a critical mediator in counteracting the cognitive deficits induced by sleep fragmentation [80,81].

#### 3.2.3. Epigenetic Regulation via Histone Lactylation

An emerging and potentially significant frontier in lactate is histone lactylation, an epigenetic modification that directly links the metabolic state to gene expression [82]. In this process, lactate modifies histone lysine residues, thereby altering chromatin structure and regulating transcription. Research has confirmed that histone lactylation is widespread across various neural cell types, including neurons and glia, and that its levels increase in response to neuronal excitation [83]. Its specific role in the context of exercise-induced cognitive enhancement remains an area of ongoing investigation. Preliminary studies suggest that exercise-induced lactate might promote hippocampal neurogenesis by regulating histone lactylation [49]. And separate findings have correlated lactate infusion with enhanced spatial memory alongside increased lactylation level. Lactate may also form a metabolic-epigenetic-metabolic circuit by regulating mitochondrial oxidative stress via lactylation [49,50].

However, it is essential to exercise caution when assigning a core status to this mechanism in the lactate nexus. Although there is evidence suggesting the neuroinflammation-regulating role of lactate through HCAR1 and regulating microglial function, which in turn affects cognitive function, through histone lactylation [84,85,86,87]. While the link between exercise and brain histone lactylation is an emerging area of research, the direct relationship between systemic exercise and brain-specific histone lactylation remains underexplored, with limited primary data. The massive influx of lactate during physical activity presents a compelling hypothesis. Exercise-induced lactate likely serves as a primary substrate for the lactylation of histones at the promoter regions of genes related to plasticity (e.g., BDNF, c-Fos). Yet, this potential mechanism for converting a transient metabolic signal from exercise into lasting transcriptional changes requires further empirical validation to determine its necessity and sufficiency in cognitive adaptations. In addition, post-translational modification of non-histone proteins by lactate represents an even newer and rarely investigated perspective that may further broaden our understanding of metabolic signalling in learning and memory [51].

## 4. Sleep: Lactate’s Nocturnal Role in Brain Restoration

Adequate sleep is crucial for cognitive function, while insufficient sleep has become a widespread public health issue, posing a serious threat to brain health. Studies have clearly shown a direct positive correlation between sleep duration and cognitive performance, particularly executive function (such as inhibitory control as measured by the Stroop test), with short sleepers performing significantly worse [88]. Further research has found that short sleep is not only associated with decreased cognitive ability but is also accompanied by elevated levels of inflammatory markers in the body, which may be one of the potential molecular mechanisms underlying its impairment of cognitive function [89]. Therefore, understanding the brain’s energy metabolism and repair processes during profound slow-wave sleep is crucial for revealing how sleep consolidates memory and maintains cognitive health. While physical activity represents a potent, acute stimulus for brain lactate, the role of lactate during sleep is equally critical, albeit functionally distinct. Sleep, and particularly slow-wave sleep (SWS), is not a passive state of rest, but an active and vital period of brain restoration, synaptic reorganisation, and memory consolidation [90]. Far from being a mere byproduct of wakeful activity that is cleared away, lactate plays a pivotal and active role in fuelling these essential nocturnal processes.

### 4.1. Lactate Dynamics Across the Sleep–Wake Cycle

Counterintuitively, brain lactate levels do not simply fall during the quietude of sleep. Instead, sophisticated measurements reveal that extracellular brain lactate levels naturally rise during the transition from wakefulness to non-REM sleep, reaching their peak during the deepest stage of sleep, SWS [31].

This rise is driven by the unique neurophysiology of SWS, which is characterised by highly synchronised, high-amplitude, low-frequency neuronal oscillations [91]. While metabolically different from the asynchronous firing of an active, wakeful brain, this synchronised activity is nevertheless an energy-demanding process that stimulates astrocytes. During SWS, it is thought that astrocytes mobilise their glycogen stores—replenished during quiet wakefulness—and ramp up glycolysis to produce and shuttle lactate to neurons. This provides a steady, reliable fuel source precisely when sleep’s most essential functions are needed [92].

### 4.2. Hypothesised Roles of Lactate in Sleep-Dependent Cognitive Functions

The overnight peak in brain lactate is intrinsically linked to one of sleep’s primary functions: memory consolidation. During wakefulness, learning strengthens synaptic connections throughout the brain. According to the synaptic homeostasis hypothesis, sleep is essential for re-normalising these connections, pruning weaker synapses, and maintaining essential ones to improve the signal-to-noise ratio, conserve energy, and prepare the brain for new learning [93].

This large-scale synaptic reorganisation is a highly energy-intensive process that requires robust ATP production [94]. Concurrently, memory consolidation involves the replay of neural activity patterns from recent experiences. This replay is driven by hippocampal sharp-wave ripples (SWRs), which are high-frequency bursts of activity during SWS that are thought to drive the transfer of memories from the temporary storage of the hippocampus to the long-term storage of the cortex [95]. The SWS-associated lactate peak serves as the preferential, on-demand energy substrate to fuel these two concurrent, energy-demanding processes. Lactate provides the necessary ATP for protein synthesis, cytoskeletal rearrangement, and cellular transport required for both large-scale synaptic downscaling and the specific synaptic strengthening driven by SWRs.

### 4.3. Potential Interactions with the Glymphatic System

The glymphatic system is the brain’s waste clearance network, responsible for flushing out metabolic byproducts, such as amyloid-beta, that accumulate during wakefulness [96]. This system functions optimally during sleep, when the brain’s interstitial space expands, allowing for the free flow of cerebrospinal fluid. While the direct link remains under active investigation, lactate metabolism is plausibly linked to glymphatic efficiency. From the perspective of energy supply, astrocytes’ maintenance of ionic gradients and cellular transport is critical for glymphatic function. The steady supply of lactate during SWS could provide the necessary ATP to power these astrocytic functions, thereby ensuring efficient waste clearance [97]. In addition, lactate and its associated metabolic changes (e.g., in pH or redox state) may act as signals that modulate the activity of aquaporin-4 (AQP4) water channels on astrocyte end-feet [98]. The correct polarisation of these channels is the primary driver of glymphatic fluid flow, suggesting that metabolic state could directly influence clearance efficiency [98].

### 4.4. Consequences of Disrupted Lactate Metabolism from Sleep Deprivation

The effects of sleep deprivation starkly illustrate the critical role of nocturnal lactate. Sleep deprivation prevents the natural, restorative rise in brain lactate [99]. This metabolic disruption is a likely contributor to the profound cognitive deficits that follow a sleepless night, particularly in areas such as learning and memory [99].

Without an adequate lactate supply, the brain is energetically starved at the precise time it needs to perform memory consolidation and synaptic reorganisation. This can impair LTP, disrupt synaptic homeostasis, and prevent the effective transfer of memories to long-term storage. Furthermore, as sleep deprivation impairs glymphatic function, it is plausible that a lack of lactate-derived energy contributes to this reduced clearance, leading to the buildup of neurotoxic waste products. Conditions such as sleep deprivation reveal that astrocytic glycogen and the lactate it produces are a critical energy reserve that couples the brain’s energy demands with its essential restorative functions [97,100]. In fact, recent work has shown that sleep deprivation activates a conserved lactate-H3K18lac-RORα axis that drives neutrophilic inflammation [101].

The model depicts the synergistic interaction between physical activity and sleep in promoting cognitive health through lactate-mediated pathways across a 24 h cycle. Moderate-to-high intensity exercise triggers a systemic surge in lactate, which enters the brain via MCT1 at the BBB. This activates the SIRT1/PGC-1α/BDNF cascade, promoting mitochondrial biogenesis and astrocytic glycogen storage, thereby priming the brain’s metabolic infrastructure. High lactate levels during exercise facilitate histone lactylation (e.g., H3K18lac) at plasticity-related gene promoters, lowering the transcriptional threshold for subsequent memory consolidation. During slow-wave sleep, brain lactate levels naturally peak. This nocturnal lactate flux fuels hippocampal sharp-wave ripples (SWRs) and memory replay while supporting waste clearance through the glymphatic system. Ultimately, these convergent mechanisms foster lifelong cognitive resilience and neuroprotection.

## 5. “Lactate Nexus” Model

The preceding chapters have established lactate’s critical but distinct roles in two fundamental physiological states: as an acute, high-volume signalling molecule during the metabolic demands of physical activity, and as a steady, crucial fuel source during the restorative processes of sleep. Viewing these roles as separate, however, overlooks the deep connection between them. This chapter goes beyond a simple comparison to introduce the “Lactate Nexus”—a combined, interactive model integrating exercise and sleep. In this framework, lactate serves as the molecular bridge that mechanistically links the cognitive benefits of both, creating a virtuous cycle of brain health.

### 5.1. The Synergistic Interplay of Exercise and Sleep: A Priming Model

The proposed priming model posits that exercise-induced lactate not only provides immediate benefits but also fundamentally recalibrates the brain’s metabolic infrastructure to optimise subsequent sleep-driven consolidation. Although direct longitudinal evidence linking exercise-lactate-sleep in a single human cohort is still developing, several independent lines of research support this synergy. For instance, acute exercise has been shown to transiently enhance the permeability of the blood–brain barrier (BBB) and increase the expression of MCT1 at the brain endothelium, effectively opening a metabolic window for the influx of muscle-derived lactate into the CNS [102]. In addition, acute bouts of exercise have been shown to upregulate the expression of MCT1 and MCT2, with this effect persisting for several hours and coinciding with the onset of subsequent sleep cycles [77]. This transport priming ensures that when lactate levels rise during slow-wave sleep (SWS), the brain has a heightened capacity to take up and utilise lactate. Furthermore, recent studies in rodents demonstrate that blocking lactate transport during a critical post-exercise window negates the sleep-dependent improvements in memory, suggesting that the metabolic legacy of exercise is indispensable for nocturnal plasticity [75].

**Figure 2:**
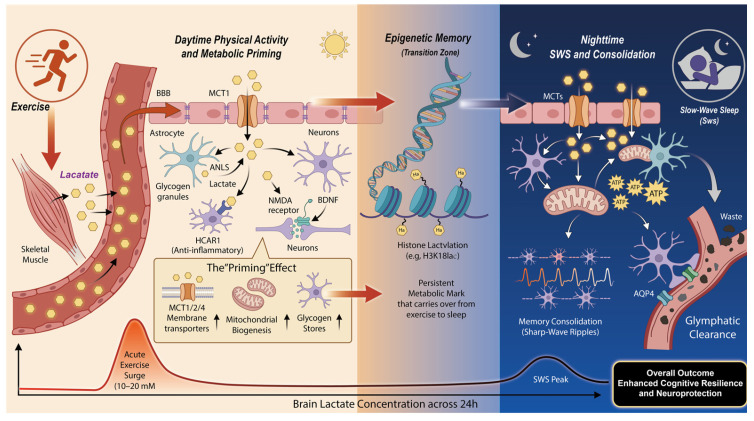
Illustration of the Lactate Nexus Model.

This priming occurs through at least three key adaptations. First, exercise can upregulate lactate transporters. Both acute and long-term exercise have been shown to increase the expression of key monocarboxylate transporters (MCTs) in the brain, including MCT1, MCT2, and MCT4 [77]. We propose that this enhanced transport capacity persists for hours post-exercise. A brain with a more efficient lactate transport system is better equipped to utilise the lactate naturally produced during slow-wave sleep, shuttling it to active neurons and glia to fuel memory consolidation more effectively. Second, lactate can increase substrate Availability. Following an exercise bout, the body works to replenish energy stores. This includes the synthesis and storage of glycogen within astrocytes. This exercise-stimulated replenishment can lead to larger glycogen reservoirs, providing a more substantial source of substrate for lactate production during the subsequent sleep period. Third, lactate can also enhance mitochondrial capacity and quality control. Exercise, particularly high-intensity interval training, is known to stimulate mitochondrial biogenesis and improve mitochondrial quality control within the hippocampus [46,48], which has been shown to depend on exercise-induced lactate release and subsequent uptake via MCTs. This adaptation means that neurons are not only receiving more lactate but are also better equipped to use it, possessing a greater capacity to convert it into ATP to power the energy-demanding processes of synaptic reorganisation and waste clearance overnight. By optimising mitochondrial efficiency and increasing mitochondrial network density, daytime exercise ensures that the brain remains energetically primed to execute the complex, ATP-intensive tasks of memory consolidation during slow-wave sleep. In this model, exercise and sleep are two phases of a single 24 h cycle. Daytime exercise serves as the stimulus that builds the machinery and stocks the fuel (upregulating MCTs, enhancing glycogen stores, and improving mitochondrial function). Nighttime sleep then capitalises on this primed, high-performance infrastructure to carry out its essential restorative and consolidative tasks more efficiently (Figure 2).

### 5.2. Histone Lactylation as an Emerging Integrative Mechanism

Beyond infrastructure, the Lactate Nexus is integrated at the epigenetic level, with histone lactylation serving as a potential key molecular mechanism that provides a lasting memory of metabolic events. This process allows the lactate signals from exercise and sleep to work in tandem. The acute, high-concentration surge of lactate during exercise acts as a powerful epigenetic signal. It drives the writing of lactylation marks on histone proteins at the promoter and enhancer regions of key plasticity-related genes (e.g., BDNF, c-Fos). This modification opens chromatin, making these genes more accessible to transcription. The subsequent period of sleep, which is the prime time for protein synthesis needed for memory consolidation, can then act on this open chromatin. We propose that the epigenetic marks induced by exercise may persist for hours, thereby lowering the transcriptional threshold required to activate these genes during memory replay (e.g., hippocampal sharp-wave ripples). The modest rise in lactate during SWS may help maintain this open state, allowing the brain to more efficiently translate the genetic blueprints flagged by exercise into the stable proteins and structural changes that form long-term memories. Thus, the two lactate signals—one high and acute from exercise, one lower and sustained during sleep—work synergistically. Exercise flags the relevant genes, and sleep provides the critical window for their expression and consolidation.

To provide a comprehensive overview of the multifaceted roles of lactate discussed throughout this review, we have synthesised key molecular targets and functional benefits of lactate across various physiological states. As summarised in Table 1, the framework delineates how lactate transitions from an acute signalling trigger during exercise to an essential fuel source and restorative mediator during sleep.

### 5.3. Gaps and Future Directions

While the “Lactate Nexus” framework offers a strong synthesis, it also reveals essential gaps in our knowledge and indicates several key pathways for future research.

A key unknown is the timing of this synergy. We need to establish the exact timeline of exercise-induced changes in brain MCT expression and histone lactylation. This prompts practical questions: Does the timing of exercise matter? For example, could exercising in the evening have a more substantial effect on sleep-related consolidation than in the morning due to the closer proximity of lactate signals? At the molecular level, we need to identify the specific genetic targets of these lactate signals. Advanced epigenetic techniques (e.g., CUT and RUN) could be employed to map and compare the histone lactylation sites in the hippocampus following exercise versus SWS. Identifying the overlap in these gene targets is a crucial step for validating this framework. Furthermore, inter-individual variability is a vital, yet underexplored factor. It is essential to examine how age, sex, APOE4 genetic risk, and fitness level influence the effectiveness of the Lactate Nexus. For instance, do older adults show a reduced lactylation response to exercise, which could account for a decreased cognitive benefit? Finally, this research must advance towards translational and imaging tools. A significant obstacle is the absence of non-invasive methods to track brain lactate dynamics in humans. Developing and validating advanced magnetic resonance spectroscopy (MRS) or similar techniques to monitor lactate transport and metabolism throughout the entire exercise–sleep–wake cycle would be invaluable for testing this framework in human populations [105].

### 5.4. Clinical Implications

The conceptualisation of the “Lactate Nexus” offers a novel theoretical framework for addressing the escalating global challenge of dementia and cognitive decline [107]. By positioning lactate as an integrative central molecular mediator, this model provides a mechanistic basis for refining non-pharmacological interventions and identifying untapped therapeutic targets. From a diagnostic perspective, the dynamic fluctuation of lactate across the sleep–wake cycle represents a potential biomarker for early cognitive vulnerability [92,106,108]. Since age-related metabolic shifts and sleep disturbances often precede clinical symptoms of neurodegeneration, monitoring these patterns through advanced imaging techniques like magnetic resonance spectroscopy (MRS) could facilitate earlier detection and more personalised risk assessment [109,110].

Beyond diagnostics, the “Lactate Nexus” underscores the importance of precision in lifestyle medicine. The proposed priming model suggests that the cognitive benefits of exercise are not merely acute but are fundamentally tied to the metabolic state of subsequent sleep. This implies that clinical recommendations should evolve to consider the optimal timing and intensity of physical activity—potentially prioritising high-intensity interval training in a way that maximises lactate-driven adaptations, such as the upregulation of MCTs and mitochondrial biogenesis, before the onset of slow-wave sleep [47,111]. Such targeted metabolic priming could be particularly transformative for older adults or those with established sleep disorders, who otherwise exhibit reduced neuroplastic responses due to impaired lactate transport mechanisms [58,112].

Furthermore, integrating lactate signalling into clinical practice provides a path to modulate chronic neuroinflammation. Given that exercise-derived lactate can shift microglia toward a neuroprotective phenotype via HCAR1 activation, lactate-based strategies may offer a potent means of counteracting the high inflammatory tone observed in individuals with short sleep duration or age-related cognitive impairment [103,104]. This shift from single-target pharmacological approaches toward leveraging endogenous, pleiotropic molecules like lactate could inspire the development of next-generation therapeutics that mimic the systemic benefits of physical activity and restorative sleep for those with limited mobility [113,114]. Ultimately, the Lactate Nexus model fosters a more holistic approach to lifelong cognitive resilience, bridging the gap between basic neurobiology and clinical application.

## 6. Conclusions

The “Lactate Nexus” framework provides a timely integration of exercise physiology and sleep science, suggesting that lactate functions not merely as a transient fuel but as a sophisticated signalling bridge that coordinates 24 h brain health. While the evidence compiled here strongly supports a role for lactate in linking daytime physical activity with nocturnal memory consolidation and glymphatic clearance, several critical considerations must be addressed to transition this hypothesis into an established neurobiological principle.

A primary limitation of the current literature is the heavy reliance on rodent models to map lactate-dependent signalling pathways. While the activation of HCAR1 and the induction of histone lactylation are robustly observed in controlled experimental settings, the direct causal relationship within the complex human exercise–sleep–cognition triad remains to be fully elucidated [115]. Future research must prioritise the use of non-invasive, high-resolution technologies, such as ^13^C-labelled magnetic resonance spectroscopy (MRS), to track real-time lactate flux in the human brain across the transition from peak physical exertion to deep slow-wave sleep. Such data would be instrumental in determining whether a metabolic threshold exists, which means that a specific intensity or volume of exercise is required to trigger the epigenetic priming and synaptic adaptations proposed in the Nexus model.

Furthermore, the translational potential of the Lactate Nexus extends to precision chronotherapy. Rather than generic recommendations for physical activity, the molecular insights provided here suggest that the timing of lactate-elevating interventions could be optimised to mitigate cognitive deficits in populations experiencing chronic sleep fragmentation. Clinical trials targeting obstructive sleep apnea or shift-work disorder should investigate whether afternoon HIIT sessions can effectively prime the brain’s metabolic infrastructure, thereby enhancing the restorative efficacy of subsequent sleep windows [116].

Finally, as we move toward clinical applications, it is crucial to delineate the boundaries of lactate’s neuroprotective effects. Future studies must carefully distinguish between the physiological lactate surges discussed in this framework and the pathologically high concentrations observed in states of stroke or neuroinflammation, which may exert neurotoxic effects. By refining our understanding of these dose- and time-dependent nuances, the Lactate Nexus may eventually serve as a cornerstone for developing targeted lifestyle interventions and pharmacological HCAR1 agonists to foster lifelong cognitive resilience.

## Figures and Tables

**Table 1:** Summary of mechanisms, molecular targets, and supporting evidence within the Lactate Nexus framework.

Physiological Context	Mechanism Category	Molecular Targets and Pathways	Functional/Cognitive Benefits	Key References
Physical Activity (Priming Phase)	Neurotrophic and Plasticity	Sirt1/PGC-1α/FNDC5/BDNF axis; TrkB receptor	Stimulates hippocampal neurogenesis; enhances LTP and synaptic strength.	[4,9,11,74,75]
	Metabolic Priming	Upregulation of MCT_1_/MCT_2_; Mitochondrial biogenesis	Enhances brain lactate uptake and utilisation capacity for subsequent sleep cycles.	[10,17,46,77,102]
	Immune Modulation	HCAR1 activation; NLRP3 inflammasome suppression	Reduces neuroinflammation; shifts microglia to a neuroprotective phenotype.	[13,34,41,72,81,103,104]
	Epigenetic Signalling	Histone lactylation (H3K18lac) at plasticity gene promoters	Lowers transcriptional thresholds for BDNF/c-Fos; establishes metabolic memory.	[49,50,82,83,87,88]
SWS—Slow-Wave Sleep (Consolidation Phase)	Energy Support	Astrocyte–Neuron Lactate Shuttle (ANLS)	Fuels hippocampal sharp-wave ripples (SWRs) and memory replay.	[15,20,28,29,92]
	Glymphatic Clearance	AQP4 water channels; Glymphatic waste clearance	Facilitates the removal of metabolic byproducts (e.g., Amyloid-β) during sleep.	[7,96,98,105,106]
Extreme Stress/Over-exercise	Neurotoxicity	ASIC channel overactivation; Mitochondrial pretender EVs	Intracellular acidosis and neuronal apoptosis; systemic-to-brain neuroinflammation.	[61,62,66,67]

Notes: BDNF, Brain-Derived Neurotrophic Factor; HCAR1, Hydroxycarboxylic Acid Receptor 1; MCT, Monocarboxylate Transporter; SWS, Slow-Wave Sleep; LTP, Long-Term Potentiation; AQP4, Aquaporin-4. Evidence strength is categorised based on the availability of causal rodent studies and human correlational data.

## Data Availability

No new data were created or analyzed in this study.

## Abbreviations

The following abbreviations are used in this manuscript:

ANLS	Astrocyte–Neuron Lactate Shuttle
AQP4	Aquaporin-4
ATP	Adenosine Triphosphate
BDNF	Brain-Derived Neurotrophic Factor
CRP	C-reactive protein
EAATs	Excitatory Amino Acid Transporters
Gln	Glutamine
GLS	Glutaminase
Glu	Glutamate
GLUT1	Glucose Transporter 1
GPR81	G protein-coupled receptor 81
GS	Glutamine Synthetase
HCAR1	Hydroxycarboxylic Acid Receptor 1
HIIT	High-Intensity Interval Training
IEGs	Immediate-Early Genes
LDH_1_	Lactate Dehydrogenase 1
LDH_5_	Lactate Dehydrogenase 5
LTP	Long-Term Potentiation
MCTs	Monocarboxylate Transporters
MRS	Magnetic Resonance Spectroscopy
NMDA	N-methyl-D-aspartate
PDH	Pyruvate Dehydrogenase
PFK-1	6-phosphofructokinase 1
PKM1	Pyruvate Kinase M1
PKM2	Pyruvate Kinase M2
SII	Systemic Immune-Inflammatory Index
SWRs	Hippocampal Sharp-Wave Ripples
SWS	Slow-Wave Sleep
TCA	Tricarboxylic Acid
VEGF	Vascular Endothelial Growth Factor
